# *MDM2* amplification is rare in gastric cancer

**DOI:** 10.1007/s00428-023-03674-8

**Published:** 2023-10-11

**Authors:** Samir Abdullazade, Hans-Michael Behrens, Sandra Krüger, Jochen Haag, Christoph Röcken

**Affiliations:** Dept. of Pathology, Christian-Albrechts-University, University Hospital Schleswig-Holstein, Arnold-Heller-Str. 3, Haus U33, D-24105 Kiel, Germany

**Keywords:** Gastric cancer, MDM2, *MDM2* amplification, MDM2 immunohistochemistry

## Abstract

The MDM2 proto-oncogene (MDM2) is a primary negative regulator of p53. The latter is frequently mutated in gastric cancer (GC). In the present study, we aimed to validate gene amplification, protein expression, and the putative tumor biological function of MDM2 in a well-characterized Western GC cohort. *MDM2* amplification and protein expression were studied in a cohort of 327 GCs by fluorescence in situ hybridization (FISH) and immunohistochemistry. Gene amplification and protein expression were correlated with diverse clinicopathological patient characteristics including patient outcome. Immunohistochemically, 97 GCs (29.7%) were categorized as MDM2 positive and 230 GCs (70.3%) as negative. An amplification of *MDM2* was found in 11 (3.4%) cases without evidence of intratumoral heterogeneity. Nine of these eleven (81.8%) cases showed MDM2 protein expression. *MDM2* amplification correlated significantly with MDM2 protein expression (*p* < 0.001). On a case-by-case analysis, *MDM2*-amplified cases showed varied histological phenotypes and were most commonly microsatellite stable; EBV, HER2, and MET negative; and FGFR2 positive. A single case harbored both, *MDM2* amplification and *TP53* mutation. *MDM2* amplification and MDM2 expression, respectively, did not correlate with overall or tumor-specific survival. Our targeted analysis of MDM2 in a well-characterized cohort of GC patients showed that *MDM2* amplification is rare, of no specific histological phenotype, and may not be always mutually exclusive with *TP53* mutations. Given the low number of cases, currently, no diagnostic or therapeutic recommendation related to *MDM2* amplification can be given for GC of Western origin.

## Introduction

Gastric cancer (GC) is the fifth most common cancer worldwide [1]. Its 5-year survival rate is still poor, ranging between 34 and 37% for men and women, respectively [2]. The vast majority are adenocarcinomas, which can be hereditary, familial, or sporadic. Common risk factors are chronic gastritis due to infection with *Helicobacter pylori*, a diet rich in salt, and lack of fresh fruits and vegetables. Less commonly, GC may be related to infection with Epstein-Barr virus (EBV). Gene polymorphisms and germline mutations modulate the individual susceptibility for GC [3].

Gastric cancer is a genetic disease affecting numerous oncogenes and tumor suppressor genes and was categorized into four molecular subtypes, i.e., chromosomal instable (CIN), genomically stable (GS), EBV-positive, and microsatellite-instable (MSI) GCs [4]. CIN-GC often has an intestinal histological phenotype according to Lauren and is associated with *TP53* mutations and activation of receptor tyrosine kinases (RTK). GS-GC often exhibits a diffuse phenotype and mutations in *CDH1* and *RHOA*, as well as *CLDN18-ARHGAP* fusion.

Using multiregional whole exome sequencing, we recently investigated the effect of somatic evolution on intratumoral heterogeneity aiming to shed light on the evolutionary biology of GC and noted that two cases harbored *MDM2* amplifications [5]. The MDM2 proto-oncogene (MDM2) is located on chromosome 12 (12q15) and encodes a protein that inhibits p53’s tumor suppressor function by blocking the transcriptional activation domain, targeting ubiquitination, and exporting to the cytoplasm [6]. *TP53* is among the most commonly mutated genes in GC accounting for 50–73% of all cases. MDM2, in turn, can be “activated” by gene amplification or promoter single nucleotide polymorphisms [7]. *MDM2* amplification and *TP53* mutations are mutually exclusive [8]. *MDM2* amplification is a common finding in malignant soft tissue tumors and infrequent in other tumor types [7–10]. Promoter polymorphism and *MDM2* amplification represent the two most extensively studied alterations. A germline single nucleotide polymorphism of the *MDM2* promoter increased MDM2 expression, increased cancer risk, and accelerated tumor progression [11]. Amplification can be assessed by comparative genomic hybridization; quantitative polymerase chain reaction; fluorescence (FISH), automated dual-color (DISH), or chromogenic in situ hybridization (CISH); and immunohistochemistry with intense diffuse nuclear staining [7, 12–15]. Few studies addressed the role of *MDM2* in GC [16–18].

Resistance to radiation and chemotherapy, to immune checkpoint inhibitor therapy, and to a RTK inhibition may be linked to *MDM2* amplification [19]. A combination of radiotherapy and MDM2-p53 inhibitor (APG-115) could boost the effect of antitumor activity *in vitro* and *in vivo* [20]. MDM2 may also predict efficacy of adjuvant fluorouracil-leucovorin-oxaliplatin (FLO) chemotherapy in resectable GC [21].

In this study, we aimed to shed further light on the prevalence and putative tumor biological function of MDM2 in GC and studied protein expression and gene amplification in a large and well-characterized cohort of Western patients with GC. We finally demonstrate that *MDM2* amplification is rare in GC, more commonly associated with microsatellite stability, FGR2 positivity, and HER2 negativity.

## Material and methods

### Statement on ethics

All procedures followed were in accordance with the ethical standards of the responsible committee on human experimentation (institutional and national) and with the Helsinki Declaration of 1964 and later versions. Informed consent for the therapeutic surgeries had been obtained from all patients. Ethical approval was obtained from the local ethical review board (D 453/10 and D 525/15) of the University Hospital Schleswig-Holstein, Kiel, Germany.

###  Patients and tumor samples

From the archive of the Department of Pathology, University Hospital Schleswig-Holstein, Campus Kiel, we retrieved all patients who have undergone partial or complete gastrectomy for adenocarcinoma of the stomach or gastroesophageal junction between 1997 and 2009. Inclusion criteria were histologically confirmed primary adenocarcinoma. Criteria of exclusion were perioperative radiotherapy or chemotherapy, and histology identified a tumor type other than adenocarcinoma. The study was based on the assumption that adenocarcinomas of the stomach and gastroesophageal junction are very alike since both show the same four molecular subtypes proposed by The Cancer Genome Atlas (TCGA) [4, 22]. The following data were retrieved from the electronic database: patient age and gender, anatomical tumor location, tumor type according to Lauren [23], tumor grade (intestinal type only), depth of local tumor invasion (pT category), number of resected lymph nodes, number of lymph nodes with metastases (pN category), lymph node ratio, presence or absence of distant metastases (pM category), tumor stage according to UICC [24], lymphatic (pL category) or vascular (pV category) invasion, and residual tumor status (pR category) [25]. Date of patient death was obtained from Epidemiological Cancer Registry of the state of Schleswig-Holstein, Germany. Hospital records and general practitioners provided the follow-up data of patients still alive. All patient-related data were pseudonymized after inclusion in the study.

### Assessing further clinicopathological characteristics

Assessments of mucin type, insulin receptor (IR) expression, FGFR2, human epidermal growth factor receptor 2 (HER2), MET, and p53 status, as well as the *RHOA*, *PIK3CA*, and *KRAS* genotype, were performed as previously described [26–33]. Modified Giemsa staining was used to evaluate infection with *H. pylori* and confirmed by polymerase chain reaction as described in detail previously [26]. BondMax detection system and EBER probe (Novocaster, Leica Microsystems GmbH, Wetzlar, Germany) were used for detecting EBV-encoded RNA based on the manufacturer’s instructions (Leica microsystems GmbH, Wetzlar, Germany) [27]. The study applied immunohistochemistry to assess microsatellite instability status using antibodies directed against MSH2, MSH6, MLH1, and PMS2 and performed subsequent molecular comparisons of the mononucleotide repeat markers NR-21, NR-24, NR-27, BAT-25, and BAT-26’s allelic profiles in the tumor for every case with absent or minimal nuclear staining [34].

### Tissue microarray construction

Formalin-fixed and paraffin-embedded tissue samples were used to generate tissue microarrays (TMA) as described previously [35]. Briefly, five separated, morphologically representative regions of the paraffin “donor” block were chosen. Tissue cylinders of 1 mm diameter were punched from these areas and precisely arrayed into a new “recipient” paraffin block using a customer-built instrument (Beecher Instruments, Silver Spring, MD, USA). After completing the block construction, 4-μm sections of the resulting tumor TMA block were cut for further analysis. Hematoxylin and eosin staining was performed to control for successful transfer of tumor tissue.

### Immunohistochemistry

Immunostaining was performed with a monoclonal mouse antibody directed against MDM2 (clone: 2A10, dilution 1:5000, Abcam, Berlin, Germany) using the BondMax Autostainer (Leica Microsystems GmbH, Wetzlar, Germany). ER2 antigen retrieval solution (20 min, Leica Microsystems GmbH, Wetzlar, Germany) was used for antigen retrieval. Staining was visualized with the Bond Polymer Refine Detection Kit (Leica Microsystems GmbH, Wetzlar, Germany). Hematoxylin served as counterstain. Immunostaining was assessed using a Zeiss microscope (Axioskop 40; Carl Zeiss AG, Oberkochen, Germany).

### Assessing immunostaining

**Fig. 1:**
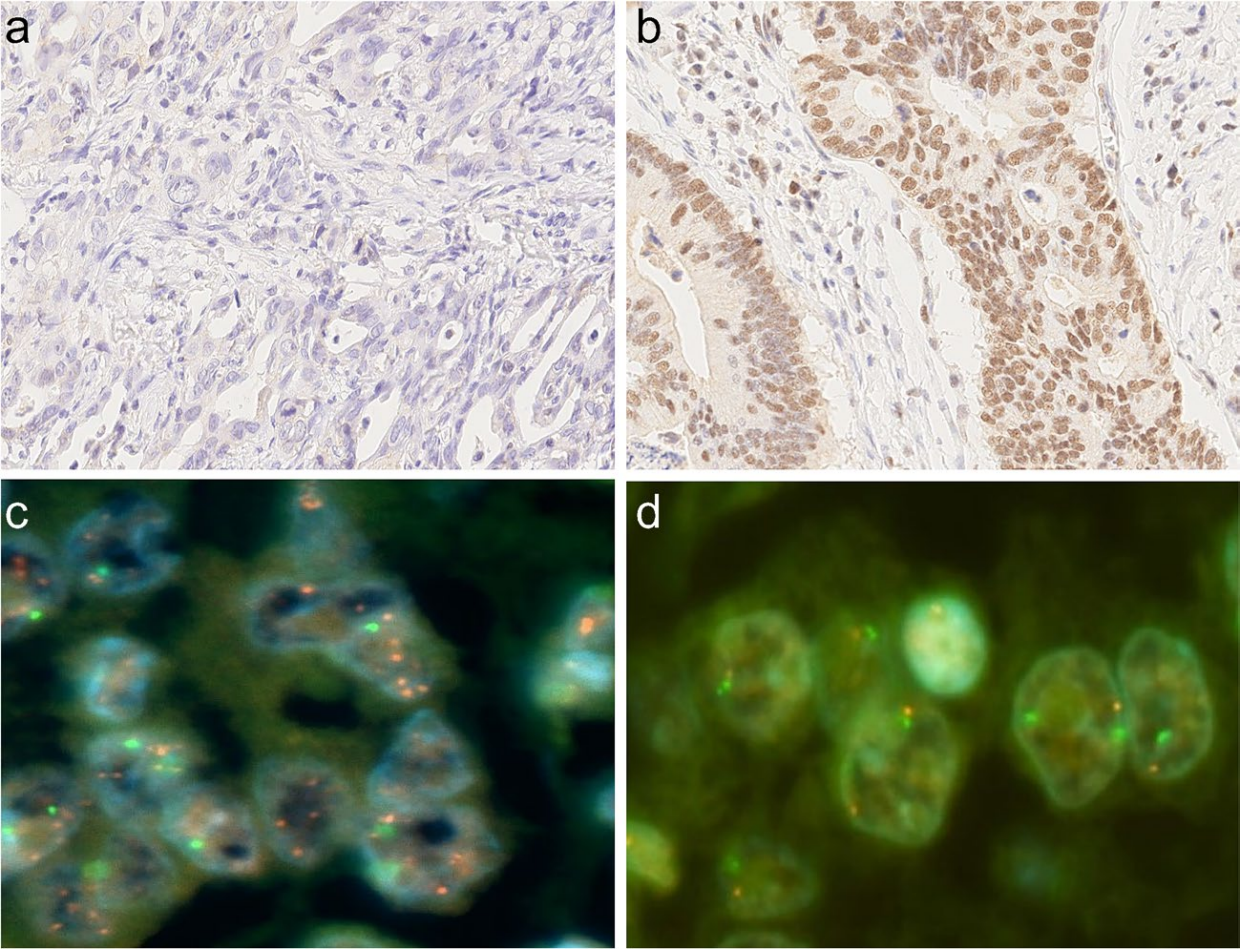
MDM2 fluorescence in situ hybridization and MDM2 immunostaining. MDM2 immunohistochemistry: case immunonegative for MDM2 (**a**) and case immunopositive for MDM2 (**b**). Anti-MDM2 antibody, hematoxylin counterstain, original magnification × 400. Case #7 with *MDM2* amplification (**c**). One case without *MDM2* amplification (**d**). Fluorescence in situ hybridization, original magnification × 1000

During evaluation of the immunostaining results, the pathologist was blinded to the clinical data. A scoring system was applied as outlined elsewhere [21]. In brief, only nuclear staining was considered and categorized as negative (0; no staining or staining of <5% of tumor cells), weak (1+), moderate (2+), or strong (3+) (Fig. 1). In addition, the percentage of stained tumor cells was recorded as 0 (<5% of tumor cells), 1+ (5–25%), 2+ (26–50%), 3+ (51–75%), and 4+ (76–100%), as previously described. Finally, a sum score was calculated combining intensity of nuclear staining and percentage of positive tumor cells. The minimum sum score was 0 and the maximum score was 7, lacking a sum score of 1.

### Fluorescence in situ hybridization

Three- to 5-μm-thick paraffin sections obtained from TMAs were used for FISH. Following dewaxing, tissue sections were re-hydrated in a descending alcohol series, incubated in 0.1 N citrate buffer 2 × 10 min, washed 2 × 2 min in dH_2_O, and incubated in 0.1 N HCl with 0.01% proteinase K for 10 min. The slides were then washed in dH_2_O for 5 min, dehydrated in descending alcohol series, dried, and incubated with 100 μl of fluorochrome-labeled probe mixture (Vysis MDM2/CEP 12 FISH Probe Kit, Abbott Molecular Inc., Des Plaines, IL 60018, USA) in a ThermoBrite hybridizer (Abbott) at 95 °C for 10 min, cooling to 37 °C and subsequently overnight. Following strict washing, dehydration, and air drying, tissue sections were finally counterstained with fluorescence mounting media containing 4′,6-diamidino-2-phenilindole (DAPI).

### Assessment of fluorescence in situ hybridization

Fluorescence in situ hybridization was evaluated with a fluorescence microscope (Axio Imager.Z2, Carl Zeiss Microscopy GmbH, Göttingen, Germany) using suitable filter sets (AHF-filter set F56-700 dual-band filter green and orange red) and the following criteria: cell nuclei had to be generally intact and distinctively visible with clear borders. The background had to be black or dark and relatively free of haziness or fluorescence particles. The signals had to be easily evaluable, distinct, oval or round shaped, compact, and bright. If these criteria were not met, the specimen was not evaluated. The number of red (*MDM2*) and green (CEP12) fluorescence signals was counted in a minimum of 30 tumor cells (Fig. 1). Subsequently, the MDM2/CEP12 ratio was calculated for each case. *MDM2* amplification was defined as MDM2/CEP12 ratio ≥ 2. A ratio <2 was classified as unamplified. *MDM2* clusters precluding exact counting of MDM2 fluorescence signals were classified as “amplified, cannot be quantitated (CBQ)”. Immunostaining of whole mount tissue sections (“full slide section”) were then additionally performed on all *MDM2*-amplified cases.

### Next-generation sequencing

Genomic DNA was extracted from formalin-fixed and paraffin-embedded tissue using the QIAamp DNA mini kit (Qiagen, Hilden, Germany) for all cases except case #4, in which no tumor DNA was available. Tissue sections were manually microdissected prior to DNA isolation to ensure a tumor cell content of higher than 80%. Library preparation was performed with AmpliSeq™ Library PLUS for Illumina using the AmpliSeq Focus Panel for Illumina and the AmpliSeq™ CD Indexes Set for Illumina® (Illumina, San Diego, USA). The quality of the libraries was assessed with a TapeStation 4150 and D1000 ScreenTapes (Agilent, Santa Clara, USA). Sequencing was done on the MiSeq System (Illumina) and sequencing data were analyzed with the Illumina VariantStudio 3.0 and the Onco CNV Caller (both Illumina). Mutations were retained if variant allele frequency (VAF) was ≥ 5%. Copy number variations were considered amplifications when total copy number was ≥6.0.

### Statistical analysis

SPSS version 25.0.0.2 (IBM Corp., Armonk, NY) was used for statistical analyses. Fisher’s exact test was used to test association between nonordinal variables. Kendall’s tau rank correlation coefficient was used to test correlation of ordinal variables. We accepted a significant level of 0.05. The Simes (Benjamini-Hochberg) procedure was used to correct for false discovery rates (FDR) [36]. All *p*-values are uncorrected. Survival curves were estimated using the Kaplan-Meier method. Differences between median survival rates were tested using the log-rank (Mantel-Cox) test.

## Results

Three hundred twenty-seven patients met all study criteria. One hundred twenty-three were female (37.6%) and 204 were male (62.4%). Median age was 68 years (range 31–92). One hundred nine (33.6%) GCs were localized in the proximal stomach (33.6%), and 215 (66.4%) in the distal stomach. According to Lauren’s classification [23], 163 (49.8%) GCs had an intestinal phenotype, 100 (30.6%) had a diffuse, and 74 (19.5%) cases were unclassifiable or mixed. Table 1 summarizes the clinicopathological patient characteristics.

**Table 1:** Clinicopathological patient characteristics

			Total		MDM2 expression				*MDM2 *amplification			
					Negative (score = 0)		Positive (score > 0)		Not amplified		Amplified	
			*n*	(%)	*n*	(%)	*n*	(%)	*n*	(%)	*n*	(%)
Total			*n*/missing									
			327	(100.0)	230	(70.3)	97	(29.7)	316	(96.6)	11	(3.4)
Gender	*n*	*p*^(1)^	327/0		327			0.617	327			0.545
Female			123	(37.6)	89	(72.4)	34	(27.6)	120	(97.6)	3	(2.4)
Male			204	(62.4)	141	(69.1)	63	(30.9)	196	(96.1)	8	(3.9)
Age group	*n*	*p*^(1)^	327/0		327			0.809	327			0.765
< 68 years			168	(51.4)	117	(69.6)	51	(30.4)	163	(97.0)	5	(3.0)
≥ 68 years			159	(48.6)	113	(71.1)	46	(28.9)	153	(96.2)	6	(3.8)
Localization	*n*	*p*^(1)^	324/3		324			0.368	324			0.048^(*)^
Proximal stomach			109	(33.6)	73	(67.0)	36	(33.0)	102	(93.6)	7	(6.4)
Distal stomach			215	(66.4)	155	(72.1)	60	(27.9)	211	(98.1)	4	(1.9)
Lauren	*n*	*p*^(1)^	327/0		327			0.466	327			0.732
Intestinal			163	(49.8)	111	(68.1)	52	(31.9)	156	(95.7)	7	(4.3)
Diffuse			100	(30.6)	74	(74.0)	26	(26.0)	98	(98.0)	2	(2.0)
Mixed			23	(7.0)	14	(60.9)	9	(39.1)	22	(95.7)	1	(4.3)
Unclassified			41	(12.5)	31	(75.6)	10	(24.4)	40	(97.6)	1	(2.4)
Mucin type	*n*	*p*^(1)^	290/37		290			0.087	290			0.886
Intestinal			76	(26.2)	60	(78.9)	16	(21.1)	73	(96.1)	3	(3.9)
Gastric			47	(16.2)	30	(63.8)	17	(36.2)	46	(97.9)	1	(2.1)
Mixed			116	(40.0)	76	(65.5)	40	(34.5)	111	(95.7)	5	(4.3)
Unclassified			51	(17.6)	40	(78.4)	11	(21.6)	50	(98.0)	1	(2.0)
Grading (intestinal type only)	*n*	*p*^(1)^	163/0		163			0.397	163			1.000
Low (G1/G2)			68	(41.7)	49	(72.1)	19	(27.9)	65	(95.6)	3	(4.4)
High (G3/G4)			95	(58.3)	62	(65.3)	33	(34.7)	91	(95.8)	4	(4.2)
pT category	*n*	*p*^(2)^	327/0		327			0.609	327			0.510
pT1a/T1b			34	(10.4)	24	(70.6)	10	(29.4)	34	(100.0)	0	(0.0)
pT2			38	(11.6)	24	(63.2)	14	(36.8)	36	(94.7)	2	(5.3)
pT3			133	(40.7)	95	(71.4)	38	(28.6)	129	(97.0)	4	(3.0)
pT4a/T4b			122	(37.3)	87	(71.3)	35	(28.7)	117	(95.9)	5	(4.1)
pN category	*n*	*p*^(2)^	326/1		326			0.523	326			0.601
pN0			90	(27.6)	63	(70.0)	27	(30.0)	88	(97.8)	2	(2.2)
pN1			46	(14.1)	34	(73.9)	12	(26.1)	44	(95.7)	2	(4.3)
pN2			60	(18.4)	45	(75.0)	15	(25.0)	58	(96.7)	2	(3.3)
pN3a/b			130	(39.9)	87	(66.9)	43	(33.1)	125	(96.2)	5	(3.8)
pM category	*n*	*p*^(1)^	327/0		327			1.000	327			1.000
pM0			264	(80.7)	186	(70.5)	78	(29.5)	255	(96.6)	9	(3.4)
pM1			63	(19.3)	44	(69.8)	19	(30.2)	61	(96.8)	2	(3.2)
UICC stage	*n*	*p*^(2)^	326/1		326			0.874	326			0.972
IA/B			48	(14.7)	35	(72.9)	13	(27.1)	47	(97.9)	1	(2.1)
IIA/B			72	(22.1)	48	(66.7)	24	(33.3)	69	(95.8)	3	(4.2)
IIIA/B/C			143	(43.9)	102	(71.3)	41	(28.7)	138	(96.5)	5	(3.5)
IV			63	(19.3)	44	(69.8)	19	(30.2)	61	(96.8)	2	(3.2)
LN ratio	*n*	*p*^(1)^	326/1		326			0.718	326			0.543
Low (<0.189)			160	(49.1)	114	(71.3)	46	(28.7)	156	(97.5)	4	(2.5)
High (≥0.189)			166	(50.9)	115	(69.3)	51	(30.7)	159	(95.8)	7	(4.2)
pL category	*n*	*p*^(1)^	312/15		312			0.326	312			0.542
pL0			153	(49.0)	111	(72.5)	42	(27.5)	149	(97.4)	4	(2.6)
pL1			159	(51.0)	107	(67.3)	52	(32.7)	152	(95.6)	7	(4.4)
pV category	*n*	*p*^(1)^	310/17		310			0.262	310			0.371
pV0			271	(87.4)	193	(71.2)	78	(28.8)	260	(95.9)	11	(4.1)
pV1			39	(12.6)	24	(61.5)	15	(38.5)	39	(100.0)	0	(0.0)
pR status	*n*	*p*^(1)^	321/6		321			0.259	321			1.000
pR0			284	(88.5)	202	(71.1)	82	(28.9)	274	(96.5)	10	(3.5)
pR1/R2			37	(11.5)	23	(62.2)	14	(37.8)	36	(97.3)	1	(2.7)
*H. pylori* status	*n*	*p*^*(*1)^	277/50		277			0.141	277			0.368
Negative			235	(84.8)	162	(68.9)	73	(31.1)	225	(95.7)	10	(4.3)
Positive			42	(15.2)	34	(81.0)	8	(19.0)	42	(100.0)	0	(0.0)
EBV status	*n*	*p*^(1)^	319/8		319			0.393	319			0.416
Negative			304	(95.3)	215	(70.7)	89	(29.3)	294	(96.7)	10	(3.3)
Positive			15	(4.7)	9	(60.0)	6	(40.0)	14	(93.3)	1	(6.7)
MSI status	*n*	*p*^(1)^	317/10		317			0.826	317			0.581
MSS			291	(91.8)	204	(70.1)	87	(29.9)	282	(96.9)	9	(3.1)
MSI			26	(8.2)	19	(73.1)	7	(26.9)	25	(96.2)	1	(3.8)
FGFR2 membranous expression	*n*	*p*^(1)^	320/7		320			0.625	320			0.542
Low			160	(50.0)	115	(71.9)	45	(28.1)	156	(97.5)	4	(2.5)
High			160	(50.0)	110	(68.8)	50	(31.3)	153	(95.6)	7	(4.4)
HER2 status	*n*	*p*^(1)^	320/7		320			1.000	320			1.000
Negative			298	(93.1)	208	(69.8)	90	(30.2)	287	(96.3)	11	(3.7)
Positive			22	(6.9)	16	(72.7)	6	(27.3)	22	(100.0)	0	(0.0)
Insulin receptor—cytoplasmatic	*n*	*p*^(1)^	299/28		299			0.614	299			0.724
Low			145	(48.5)	104	(71.7)	41	(28.3)	142	(97.9)	3	(2.1)
High			154	(51.5)	106	(68.8)	48	(31.2)	149	(96.8)	5	(3.2)
Insulin receptor—membranous	*n*	*p*^(1)^	299/28		299			0.044^(*)^	299			0.482
Low			158	(52.8)	119	(75.3)	39	(24.7)	155	(98.1)	3	(1.9)
High			141	(47.2)	91	(64.5)	50	(35.5)	136	(96.5)	5	(3.5)
Insulin receptor—vascular	*n*	*p*^(1)^	299/28		299			0.130	299			0.488
Low			144	(48.2)	95	(66.0)	49	(34.0)	139	(96.5)	5	(3.5)
High			155	(51.8)	115	(74.2)	40	(25.8)	152	(98.1)	3	(1.9)
MET status	*n*	*p*^(1)^	317/10		317			0.033^(*)^	317			0.199
Negative			293	(92.4)	212	(72.4)	81	(27.6)	284	(96.9)	9	(3.1)
Positive			24	(7.6)	12	(50.0)	12	(50.0)	22	(91.7)	2	(8.3)
*BRAF* genotype	*n*	*p*^(1)^	321/6		321			0.299	321			1.000
Wildtype			320	(99.7)	225	(70.3)	95	(29.7)	309	(96.6)	11	(3.4)
Mutated			1	(0.3)	0	(0.0)	1	(100.0)	1	(100.0)	0	(0.0)
*KRAS* genotype	*n*	*p*^(1)^	321/6		321			0.358	321			1.000
Wildtype			308	(96.0)	214	(69.5)	94	(30.5)	297	(96.4)	11	(3.6)
Mutated			13	(4.0)	11	(84.6)	2	(15.4)	13	(100.0)	0	(0.0)
*MDM2* amplification	*n*	*p*^(1)^	327/0		327			<0.001				
Not amplified					228	(72.2)	88	(27.8)				
Amplified					2	(18.2)	9	(81.8)				
*PIK3CA* genotype	*n*	*p*^(1)^	321/6		321			0.291	321			1.000
Wildtype			303	(94.4)	210	(69.3)	93	(30.7)	292	(96.4)	11	(3.6)
Mutated			18	(5.6)	15	(83.3)	3	(16.7)	18	(100.0)	0	(0.0)
p53 expression	*n*	*p*^(1)^	319/8		319			0.713	319			1.000
Low			157	(49.2)	109	(69.4)	48	(30.6)	152	(96.8)	5	(3.2)
High			162	(50.8)	116	(71.6)	46	(28.4)	156	(96.3)	6	(3.7)
*TP53* genotype	*n*	*p*^(1)^	78/249		78			0.266	78			1.000
Wildtype			57	(73.1)	43	(75.4)	14	(24.6)	54	(94.7)	3	(5.3)
Mutated			21	(26.9)	13	(61.9)	8	(38.1)	20	(95.2)	1	(4.8)
*RHOA* genotype	*n*	*p*^(1)^	280/47		280			1.000	280			1.000
Wildtype			269	96.1	184	(68.4)	85	(31.6)	261	(97.0)	8	(3.0)
Mutated			11	3.9	8	(72.7)	3	(27.3)	11	(100.0)	0	(0.0)
Overall survival (months)		*p*^(3)^	319/8		319		0.288		319		0.727	
Total/events/censored			319/252/67		225/178/47		94/74/20		308/243/65		11//2	
Median survival			14.1 ± 1.2		16.0 ± 1.6		12.5 ± 2.0		14.1 ± 1.2		16.8 ± 5.1	
95% C.I.			11.6–16.5		12.8–19.2		8.5–16.5		11.6–16.5		6.8–26.7	
Tumor-specific survival (months)		*p*^(3)^	296/31		296		0.890		296		0.724	
Total/events/censored			296/201/95		213/149/64		83/52/31		289/197/92		7/4/3	
Median survival			16.5 ± 1.4		16.7 ± 1.7		15.6 ± 2.2		16.5 ± 1.5		16.8 ± 17.0	
95% C.I.			13.7–19.3		13.3–20.1		11.3–19.9		13.6–19.4		0–50.2	

^(1)^Fisher’s exact test; ^(2)^Kendall’s tau test; ^(3)^log-rank test; ^(*)^not significant after multiple testing correction

### Expression of MDM2 in gastric cancer

First, we examined the expression of MDM2 in GC by immunohistochemistry (Fig. 1a, b). Nuclear staining of MDM2 was found in 97 (29.7%) cases. Weak immunostaining (MDM2-1+) was observed in 67 (20.5%) cases, moderate (MDM2-2+) in 29 (8.9%), and strong (MDM2-3+) in 1 (0.3%). No immunostaining was found in 230 (70.3%) GCs. The percentage of the immunostained tumor area varied for all three staining intensities (weak to strong), ranging from 5 to 100%, with scores ranging from 1 to 4. The sum score was 2 in 58 (17.7%) cases, 3 in 20 (6.1%), 4 in 9 (2.8%), 5 in 8 (2.4%), and 6 in 2 (0.6%). There was no case with a sum score 7.

### MDM2 amplification in gastric cancer

Next, we explored the amplification of *MDM2* in GC by FISH (Fig. 1c, d). Eleven cases (3.4%) showed amplification of the *MDM2*. The mean *MDM2*/chromosome 12 ratio was 4.6 (range 3–8). In order to assess intratumoral heterogeneity and to avoid sampling error, we repeated FISH analysis using whole-mount tissue sections obtained from the eleven *MDM2*-amplified cases. *MDM2* amplification was homogeneous in all these cases without evidence of intratumoral heterogeneity.

**Fig. 2:**
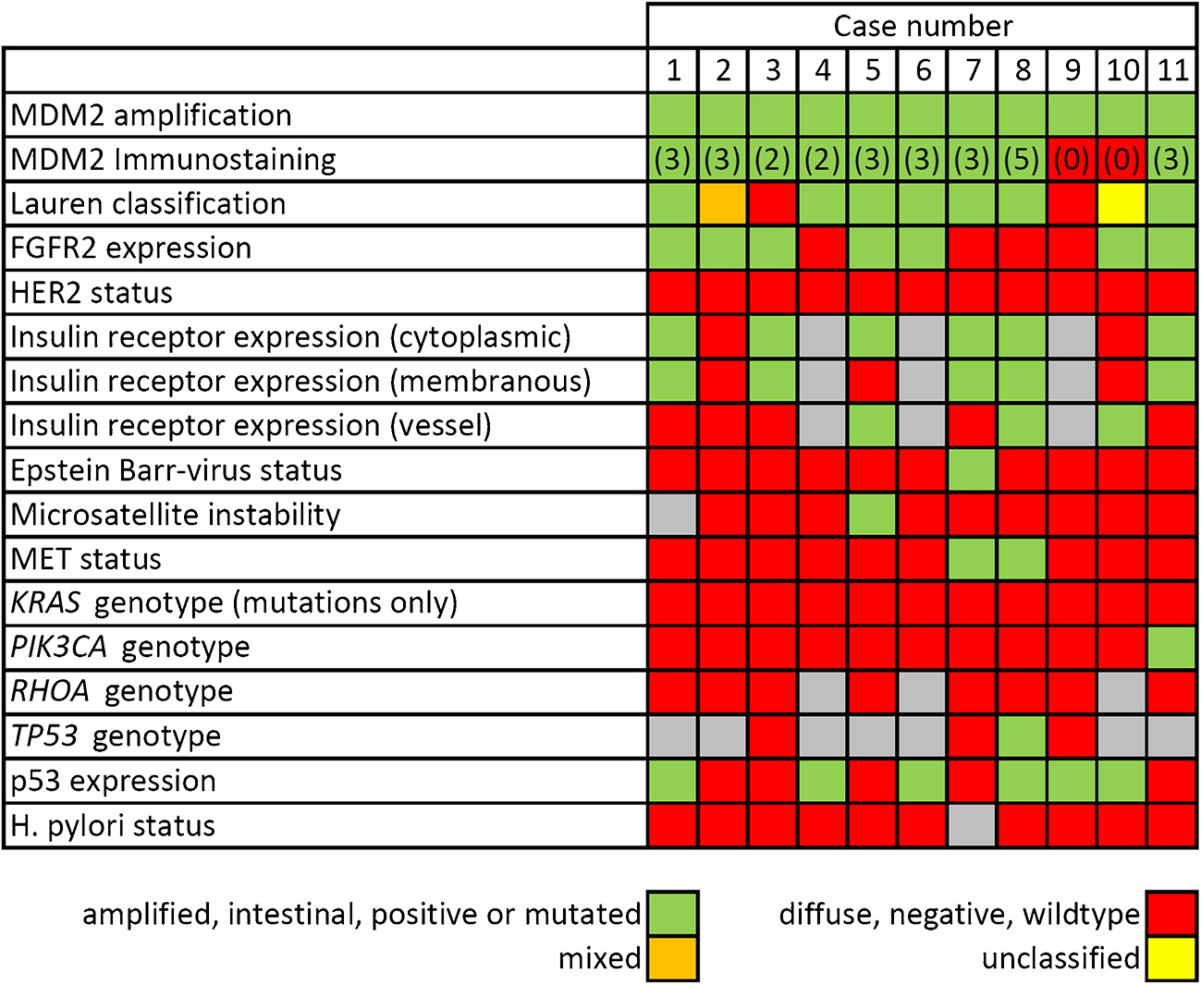
Case-wise summary of the phenotypic/genotypic co-alterations associated with *MDM2* amplification. Green: cases with *MDM2* amplification, intestinal phenotype, immunohistochemical reactivity, or mutated; red: cases without immunohistochemical reactivity, wildtype, or diffuse phenotype; gray: not available; yellow: unclassified; and orange: cases with mixed phenotype. Numbers in brackets document the sum score of MDM2 immunostaining

We then correlated *MDM2* amplification with MDM2 expression. The MDM2 protein expression (sum score 2–6) was absent in 228 (72.2%) GCs without gene amplification, while *MDM2*-amplified cases expressed MDM2 protein in 9 of 11 cases (81.8%). In these cases, weak to strong nuclear immunopositivity was found and the sum score ranged from 2 to 5. Two cases with *MDM2* amplification had no MDM2 protein expression (18.2%) (Fig. 2). Thus, MDM2 expression was significantly more common in *MDM2*-amplified cases (*p* < 0.001).

### Phenotype of MDM2-amplified gastric cancers

Next, we explored the correlation of genotype with phenotype and reviewed the histology all *MDM2-*amplified cases. Seven were of an intestinal, two of a diffuse, and one of each mixed or unclassifiable according to Lauren (Fig. 2).

### Correlation with clinicopathological patient characteristics

In order to explore the putative tumor biological significance, we correlated both, MDM2 expression and *MDM2* amplification, with diverse clinicopathological patient characteristics. Following dichotomization of the MDM2 expression at the median immunoscore (0 vs. >0), MDM2-positive tumors were more commonly MET positive (*p*=0.033) and showed more commonly a membranous expression of the insulin receptor (*p*=0.044). No other clinicopathological patient characteristic correlated with the MDM2 expression. Following a separate analysis of intestinal type GCs, no correlation was found between MDM2 expression and any clinicopathological patient characteristic (data not shown).

With regard to copy number variation, it was interesting to note that *MDM2*-amplified GCs were commonly localized in the proximal stomach, were all negative for *H. pylori* and HER2, and were a *KRAS*, *PIK3CA*, and *RHOA* wildtype (Fig. 2). Interestingly, 9 (82%) cases were negative for MET and 10 (91%) for EBV. Microsatellite instability was noted in a single, *MDM2*-amplified case, while FGFR2 positivity was found in 7 (64%) *MDM2*-amplified cases (Fig. 2). Due to low case numbers, none of these findings was statistically significant.

### Next-generation sequencing

To further explore the genotype of *MDM2*-amplified cases and search for putative druggable targets, tumor DNA of ten cases (case #1–3 and case #5–11) was forwarded to next-generation sequencing using the Oncomine™ Focus Assay, which covers hot spot mutations, copy number variations, and fusions of 52 different genes. As shown in Table 2, an amplification of *CDK4* was found in two cases, among which one also showed an amplification of *KRAS* and *ERBB3*. No other alteration matched with *MDM2* amplification. Two cases (# 6 and #10) were not assessable due to low DNA quality. Noteworthy, NGS did not detect the *MET* amplification in case #8 and #9 due to intratumoral heterogeneity of MET amplification [29].

**Table 2:** Next-generation sequencing (NGS) with Oncomine™ Focus Assay identified only few copy number variations and mutations in nine cases with *MDM2* amplification. In two cases, DNA quality did not allow NGS analysis

Case number	Copy number variation	Chromosome	Fold change	Mutation	HGVSc	HGVSp	VAF	Read depth
1	*CDK4*	*12*	6.5	*ERBB4*	NM_005235.2:c.2103delT	NP_005226.1:p.Ser701ArgfsTer13	5.5	5555
2	None detected			None detected				
3	None detected			*AR*	NM_000044.3:c.2608-1G>T	Splice variant	5	8385
4	Not assessed			Not assessed				
5	*CDK4*	*12*	6.0	*NRAS*	NM_002524.4:c.38G>A	NP_002515.1: p.Gly13Asp	32.8	1924
	*ERBB3*	*12*	6.0					
	*KRAS*	*12*	6.0					
6	Not assessable*			Not assessable*				
7	None detected			*CTNNB1*	NM_001904.3:c.134C>T	NP_001895.1: p.Ser45Phe	8.7	381
8	None detected			*FGFR1*	NM_001174067.1:c.842G>A	NP_001167538.1: p.Arg281Gln	50.8	1338
9	None detected			None detected				
10	Not assessable*			Not assessable*				
11	None detected			PIK3CA*	NM_006218.2:c.103G>A	NP_006209.2: p.Glu35Lys	6	499
				PIK3CA*	NM_006218.2:c.85G>A	NP_006209.2: p.Gly29Arg	5.2	499

*Not assessable due to low DNA quality

### Prognostic significance

**Fig. 3:**
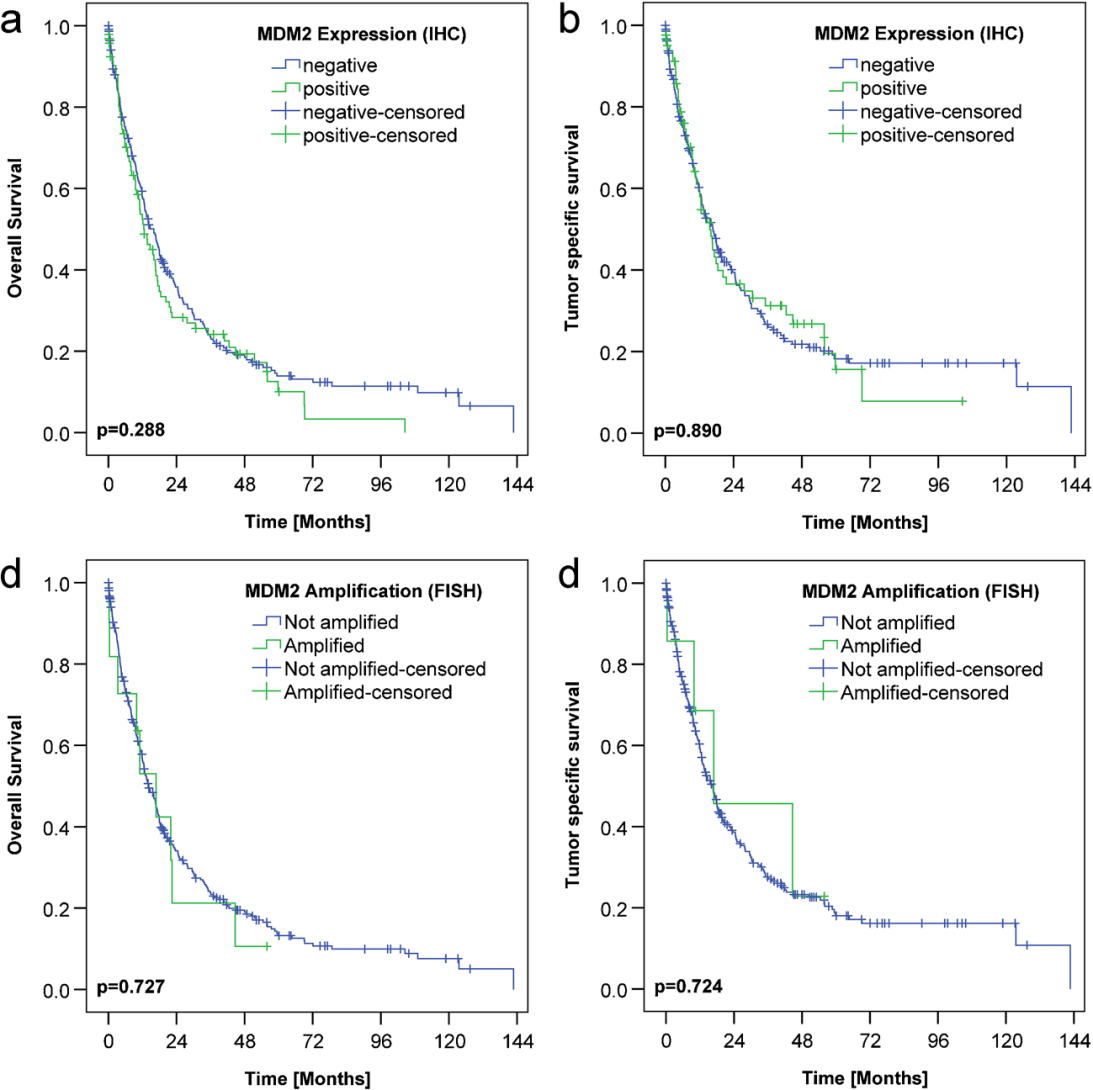
Kaplan-Meier curves of patient overall survival and tumor-specific survival using expression of MDM2 in the immunohistochemical examination (IHC) and *MDM2* amplification using fluorescence in situ hybridization (FISH). **a**, **b** Patients’ overall survival and tumor-specific survival according to MDM2 expression; negative, no expression; positive, cases with a sum score 2–6. **c**, **d** Patients’ overall survival and tumor-specific survival according to FISH, amplified, not amplified

No significant difference was found in the overall and tumor-specific survival between *MDM2*-amplified and nonamplified cases nor between cases with or without MDM2 expression (Fig. 3).

## Discussion

To the best of our knowledge, our study is the single most extended analysis of *MDM2* amplification in GC of White patients. Overall, amplification of *MDM2* was rare accounting only for 3.4% of our cases, a prevalence supported by previous findings. *MDM2* amplification was present in 1.6% of TCGA cohort and 5.5% of the cohort studied by Kato et al. [7, 10]. Among the 1114 cases with an adenocarcinoma of the esophagus, gastroesophageal junction, or stomach documented in the cBioPortal database (search date 17 September 2023), 72 (6.5%) cases harbored an *MDM2* amplification and only 8 (0.7%) a mutation. Structural variants have not been reported [37]. Amplification rates may not be uniform across tumor types. Günther et al. demonstrated *MDM2* amplification by Southern blot analysis in 18 of 43 (41.8%) advanced-stage GCs, commonly presenting with a diffuse growth pattern [17]. However, in our series, no evidence of intratumoral heterogeneity for *MDM2* amplification was found and we studied a much larger patient cohort.

*MDM2* amplification may lead to (over)expression of MDM2, although other mechanisms have been reported, e.g., germline single nucleotide polymorphisms. We were able to demonstrate a significant correlation between *MDM2* amplification and protein expression. *MDM2*-amplified cases expressed MDM2 in 9 cases (81.8%) with gene amplification and MDM2 expression was significantly more common in *MDM2*-amplified cases. However, eighty-eight GCs were classified as MDM2 positive by immunohistochemistry without *MDM2* amplification. Furthermore, the MDM2 protein expression was absent in two tumors with *MDM2* amplification. Similar findings were reported from Cordon-Cardo et al. in soft tissue tumors. Twenty-seven percent of the sarcomas with MDM2 expression lacked *MDM2* amplification, and vice versa 45% of the tumors with gene amplification lacked MDM2 expression [38]. A putative explanation might be mRNA splicing, which leads to different forms of the MDM2 protein, thereby prohibiting immunohistochemical detection. Marchetti et al. in the same sense indicated that the utilized antibodies might identify varied epitopes only, making immunohistological confirmation sometimes impossible [39]. They observed 21 cases without *MDM2* amplification but MDM2 protein immunohistochemical reactivity, which in few cases was quite widespread [39]. Thus, gene amplification does not always correlate with protein expression and lack of immunostaining does not exclude *MDM2* amplification. Molecular pathological analysis of *MDM2* may give higher specificity and sensitivity compared with immunohistochemistry [40, 41].

Next, we tested the hypothesis that *MDM2*-amplified and/or MDM2-positive GCs harbor a specific molecular or clinical phenotype. While the overall number of *MDM2*-amplified GCs was low, it was interesting to note that these tumors frequently were of *KRAS*, *PIK3CA,* and *RHOA* wildtype; microsatellite stable; *H. pylori* and EBV negative; HER2 and MET negative; and positive for FGFR2 (Fig. 3). In addition, a single case showed both *MDM2* amplification and *TP53* mutation. Thus, it seems that MDM2 amplification and *TP53* mutation are not mutually exclusive in all cases with GC. Supporting our findings, no significant difference in *TP53* mutation frequency and *MDM2* amplification status was found in esophageal carcinomas [42]. On the contrary, *MDM2* alterations are mutually exclusive with *TP53* mutations in sarcomas. However, further studies on larger case series are warranted to substantiate these observations. At least in our series, no correlation was found between p53 expression and *MDM2* amplification.

*MDM2* amplification co-altered with *TP53* indicates a noncanonical, p53-independent role of MDM2 in tumor biology, which was apparent in breast cancer [39]. MDM2 facilitates angiogenesis as one of its suggested noncanonical effects [43]. The distribution of p53 alterations in identifying tumor type contrasted with the distribution of *MDM2* amplification. According to Zhou et al., MDM2 regulates vascular endothelial growth factor translation, and it is active contributor to increased cancer cell growth and angiogenesis [44].

In summary, our study on a large cohort of GCs of Western origin shows that *MDM2* amplification is rare in GC and more common in intestinal type GC. However, on a case-by-case analysis, intriguing findings were made, such as that *MDM2*-amplified cases were most commonly microsatellite stable; EBV, HER2, and MET negative, and FGFR2 positive.

## Conclusion

Whole exome sequencing is becoming a mainstay of precision medicine and provides massive data on tumor genetics. These data are increasingly used to tailor patient treatment, particularly in the palliative setting: molecular tumor boards heavily rely on published data about the putative tumor biological significance of mutated target genes. However, there is a growing gap between the availability of genetic data and validation studies exploring the putative tumor biological significance of the respective gene product. To fill this gap of information, we extended our previous genetic research on nine GCs, two of which harbored an *MDM2* amplification [5]. The targeted analysis of MDM2 in a well-characterized cohort of GC patients showed that *MDM2* amplification is rare, more commonly of intestinal phenotype, although not exclusively, and may not be mutually exclusive with *TP53* mutations. Given the low number of cases, currently, no diagnostic or therapeutic recommendation related to *MDM2* amplification can be given for GC of Western origin.

## References

[1] Sung H, Ferlay J, Siegel RL, Laversanne M, Soerjomataram I, Jemal A, Bray F (2021). Global cancer statistics 2020: GLOBOCAN estimates of incidence and mortality worldwide for 36 cancers in 185 countries. CA Cancer J Clin.

[2] Robert-Koch-Institut (2021). Krebs in Deutschland für 2017/2018.

[3] Röcken C, Warneke V (2012). Molekulare Pathologie des Magenkarzinoms. Pathologe.

[4] The Cancer Genome Atlas Research Network (2014). Comprehensive molecular characterization of gastric adenocarcinoma. Nature.

[5] Röcken C, Amallraja A, Halske C (2021). Multiscale heterogeneity in gastric adenocarcinoma evolution is an obstacle to precision medicine. Genome Med.

[6] Roth J, Dobbelstein M, Freedman DA, Shenk T, Levine AJ (1998). Nucleo-cytoplasmic shuttling of the hdm2 oncoprotein regulates the levels of the p53 protein via a pathway used by the human immunodeficiency virus rev protein. EMBO J.

[7] Oliner JD, Saiki AY, Caenepeel S (2016). The role of MDM2 amplification and overexpression in tumorigenesis. Cold Spring Harb Perpect Med.

[8] Momand J, Jung D, Wilczynski S, Niland J (1998). The MDM2 gene amplification database. Nucleic Acids Res.

[9] Oliner J, Kinzler K, Meltzer P, George DL, Vogelstein B (1992). Amplification of a gene encoding a p53-associated protein in human sarcomas. Nature.

[10] Kato S, Ross JS, Gay L, Dayyani F, Roszik J, Subbiah V, Kuryrock R (2018). Analysis of MDM2 amplification: next-generation sequencing of patients with diverse malignancies. JCO Precis Oncol.

[11] Bond GL, Hu W, Bond EE, Robins H, Lutzker SG, Arva NC, Bargonetti J, Bartel F, Taubert H, Wuerl P, Onel K, Yip L, Hwang SJ, Strong LC, Lozano G, Levien AJ (2004). A single nucleotide polymorphism in the MDM2 promoter attenuates the p53 tumor suppressor pathway and accelerates tumor formation in humans. Cell.

[12] Coindre JM, Pedeutour F, Aurias A (2010). Well-differentiated and dedifferentiated liposarcomas. Virchows Arch.

[13] Kobayashi A, Sakuma T, Fujimoto M, Jimbo N, Hirose T (2019). Diagnostic utility and limitations of immunohistochemistry of p16, CDK4, and MDM2 and automated dual-color in situ hybridization of MDM2 for the diagnosis of challenging cases of dedifferentiated liposarcoma. Appl Immunohistochem Mol Morphol.

[14] Jimbo N, Komatsu M, Itoh T, Hirose T (2019). MDM2 dual-color in situ hybridization (DISH) aids the diagnosis of intimal sarcomas. Cardiovasc Pathol.

[15] Mardekian SK, Solomides CC, Gong JZ, Peiper SC, Wang Z, Bajaj R (2015). Comparison of chromogenic in situ hybridization and fluorescence in situ hybridization for the evaluation of MDM2 Amplification in adipocytic tumors. J Clin Lab Anal.

[16] Blok P, Craanen ME, Dekker W, Offerhaus GJA, Tytgat GNJ (1998). No evidence for functional inactivation of wild type p53 protein by mdm2 overexpression in gastric carcinogenesis. J Pathol.

[17] Günther T, Schneider-Stock R, Häckel C, Kasper HU, Pross M, Hackelsberger A, Lippert H, Roessner A (2000). Mdm2 gene amplification in gastric cancer correlation with expression of Mdm2 protein and p53 alterations. Mod Pathol.

[18] Bartpho TS, Wattanawongdon W, Tongtawee T (2021). The mouse double minute 2 polymorphism is associated with both decreased p53 expression and poor clinicopathological outcomes of gastric cancer. J Can Res Ther.

[19] Hou H, Sun D, Zhang X (2019). The role of MDM2 amplification and overexpression in therapeutic resistance of malignant tumors. Cancer Cell Int.

[20] Yi H, Yan X, Luo Q, Yuan L, Li B, Pan W, Zhang Y, Chen H, Wang J, Zhang Y, Zhai Y, Qiu M, Yang D (2018). A novel small molecule inhibitor of MDM2-p53 (APG-115) enhances radiosensitivity of gastric adenocarcinoma. J Exp Clin Can Res.

[21] Ye Y, Li X, Yang J, Miao S, Chen Y, Xia X, Wu X, Zhang J, Zhou Y, He S, Tan Y, Qiang F, Li G, Roe OD, Zhou J (2013). MDM2 is a useful prognostic biomarker for resectable gastric cancer. Cancer Sci.

[22] The Cancer Genome Atlas Research Network (2017). Integrated genomic characterization of esophageal carcinoma. Nature.

[23] Lauren P (1965). The two histologic main types of gastric carcinoma: diffuse and so-called intestinal-type carcinoma. Acta Pathol Microbiol Scand.

[24] Brierley J, Gospodarowicz MK, Wittekind C (2017). TNM classification of malignant tumours.

[25] Hermanek P, Wittekind C (1994). Residual tumor (R) classification and prognosis. Semin Surg Oncol.

[26] Warneke VS, Behrens HM, Haag J, Balschun K, Böger C, Becker T, Ebert MPA, Lordick F, Röcken C (2013). Prognostic and putative biomarkers of gastric cancer for personalized medicine. Diagn Mol Pathol.

[27] Böger C, Krüger S, Behrens HM, Bock S, Haag J, Kalthoff H, Röcken C (2017). Epstein-Barr virus associated gastric cancer reveals intratumoral heterogeneity of PIK3CA mutations. Ann Oncol.

[28] Warneke VS, Behrens HM, Böger C, Becker T, Lordick F, Ebert MPA, Röcken C (2013). Her2/neu testing in gastric cancer: evaluating the risk of sampling errors. Ann Oncol.

[29] Metzger ML, Behrens HM, Böger C, Haag J, Krüger S, Röcken C (2016). MET in gastric cancer – discarding a 10% cutoff rule. Histopathology.

[30] Schoop I, Maleki SS, Behrens HM, Krüger S, Haag J, Röcken C (2020). p53 immunostaining cannot be used to predict TP53 mutations in gastric cancer: results from a large Central European cohort. Human Pathol.

[31] Schrumpf T, Behrens HM, Haag J, Krüger S, Röcken C (2022). FGFR2 overexpression and compromised survival in diffuse-type gastric cancer in a large central European cohort. PLoS ONE.

[32] Röcken C, Behrens HM, Böger C, Krüger S (2016). Clinicopathological characteristics of RHOA mutations in a Central European gastric cancer cohort. J Clin Pathol.

[33] Heckl SM, Wiesener V, Behrens HM, Ulase D, Krüger S, Röcken C (2019). The expression of the insulin receptor in gastric cancer correlates with the HER2 status and may have putative therapeutic implications. Gastric Cancer.

[34] Mathiak M, Warneke VS, Behrens HM, Haag J, Böger C, Krüger S, Röcken C (2017). Clinicopathologic characteristics of microsatellite instable gastric carcinomas revisited: urgent need for standardization. Appl Immunhistochem Mol Morphol.

[35] Kononen J, Bubendorf L, Kallioniemi A, Barlund M, Schraml P, Leighton S, Torhorst J, Mihatsch MJ, Sauter G, Kallioniemi OP (1998). Tissue microarrays for high-throughput molecular profiling of tumor specimens. Nat Med.

[36] Simes RJ (1986). An improved Bonferroni procedure for multiple tests if significance. Biometrika.

[37] Gao J, Aksoy BA, Dogrusoz U, Dresdner G, Gross B, Sumer SO, Sun Y, Jacobsen A, Sinha R, Larsson E, Cerami E, Sander C, Schultz N (2013). Integrative analysis of complex cancer genomics and clinical profiles using the cBioPortal. Sci Signal.

[38] Cordon-Cardo C, Latres E, Drobnjak M, Oliva MR, Pollack D, Woodruff VM, Marechal V, Chen J, Brennan MF, Levine AJ (1994). Molecular abnormalities of MDM2 and p53 genes in adult soft tissue sarcomas. Cancer Res.

[39] Marchetti A, Buttitta F, Girlando S, Dalla Palma P, Pellegrini S, Fina P, Doglioni C, Bevilacqua G, Barbareschi M (1995). MDM2 gene alterations and MDM2 protein expression in breast carcinomas. J Pathol.

[40] Machado I, Vargas AC, Maclean F, Llombart-Bosch A (2022). Negative MDM2/CDK4 immunoreactivity does not fully exclude MDM2/CDK4 amplification in a subset of atypical lipomatous tumor/well differentiated liposarcoma. Pathol Res Pract.

[41] Weaver J, Sowns-Kelly E, Goldblum JR, Turner S, Kulkarni S, Tubbs RR, Rubin BP, Skacel M (2008). Fluorescence in situ hybridization for MDM2 gene amplification as a diagnostic tool in lipomatous neoplasms. Mod Pathol.

[42] Michalk M, Meinrath J, Künstlinger H, Koitzsch U, Drebber U, Merkelbach-Bouse S, Bollschweller E, Kloth M, Hartmann W, Hölschner A, Quaas A, Grimminger PP, Odenthal M (2016). MDM2 gene amplification in esophageal carcinoma. Oncology Rep.

[43] Kim ES, Shohet JM (2015). Reactivation of p53 via MDM2 inhibition. Cell Death Dis.

[44] Zhou S, Gu L, He J, Zhang H, Zhou M (2011). MDM2 regulates vascular endothelial growth factor mRNA stabilization in hypoxia. Mol Cell Biol.

